# Organochlorine pesticides and polychlorinated biphenyls along an east-to-west gradient in subtropical North Atlantic surface water

**DOI:** 10.1007/s11356-016-7429-z

**Published:** 2016-08-18

**Authors:** Gerhard Lammel, Alejandro Spitzy, Ondřej Audy, Sabine Beckmann, Garry P. Codling, Lisett Kretzschmann, Petr Kukučka, Irene Stemmler

**Affiliations:** 1 Multiphase Chemistry Department, Max Planck Institute for Chemistry, Mainz, Germany; 2 Research Centre for Toxic Compounds in the Environment, Masaryk University, Brno, Czech Republic; 3 Centre for Earth System Research and Sustainability, Institute for Geology, University of Hamburg, Hamburg, Germany; 4 Max Planck Institute for Meteorology, Hamburg, Germany; 5 Federal Maritime and Hydrographic Agency (BSH), Hamburg, Germany; 6 Man-Technology-Environment Research Centre, Örebro University, Örebro, Sweden

**Keywords:** Ocean pollution, Persistent organic pollutants, Trace analytical chemistry, Environmental fate

## Abstract

Despite the fact that most persistent toxic substances have hardly been primarily emitted for several decades, their concentrations are only slowly decreasing in the global oceans. Surface seawater samples were collected along a 38°–24° N/28°–67° W transect in the subtropical North Atlantic Ocean. While the concentration levels of hexachlorobenzene (2.1–6.1 pg L^−1^), dichlorodiphenyltrichloroethane (DDT, up to 2.1 pg L^−1^) and polychlorinated biphenyls (PCB, 10.8–24.9 pg L^−1^) were in the same range as observed earlier in the North Atlantic, hexachlorocyclohexane (HCH, 90–627 pg L^−1^) was found elevated, partly also relative to previous measurements in the same sea region. Hereby, the ratio α*-*HCH/γ-HCH was very low, 0.09–0.13. Chlordane and endosulfan were found in the range <3.0–11.1 and <5.8–8.8 pg L^−1^ respectively. DDT metabolites, endrin and related pesticides were found below quantification limits. Spatial pollution patterns in surface seawaters seem to be determined by atmospheric and oceanic transport patterns, rather than by mixing and air-sea equilibrium. The comparison with global multicompartment chemistry-transport model predictions of surface seawater levels indicate underestimated degradation of PCBs and overestimated emissions of endosulfan.

**Electronic supplementary material:**

The online version of this article (doi:10.1007/s11356-016-7429-z) contains supplementary material, which is available to authorized users.

## Introduction

Persistent pesticides are nowadays banned in most countries. They have hardly been primarily emitted since several decades, but their concentrations may be sustained by secondary emissions from oceans and land surfaces (Lammel and Stemmler 2012), only slowly decreasing in the global oceans (Lohmann et al. 2007). Their concentrations might even re-rise in the deep sea (Stemmler and Lammel 2013). Decreasing concentrations in surface waters may be in response to decreasing atmospheric levels as was observed and predicted for the insecticides hexachlorocyclohexane (HCH) and dichlorodiphenyltrichloroethane (DDT) (Jantunen and Bidleman 1998; Stemmler and Lammel 2009), or reflecting vertical transports in the ocean (Lohmann et al. 2007; Nizzetto et al. 2010). The globally most important substitute for DDT, endosulfan, has been heavily used in agriculture since the 1970s and banned as a persistent organic pollutant (POP) in countries since 2013. However, its spatial and temporal trends in the global marine environment are not established (Weber et al. 2010).

Unlike on continents of the northern hemisphere and the Arctic, no systematic chemical monitoring of the oceans is in place at all (UNEP, 2003; Lohmann and Muir 2010). Current knowledge on the distribution of organic pollutants in the ocean relies on rare campaign-based measurements. For example in seawater of the open Atlantic, no measurement of *p,p′*-DDT (the main isomer of the technical mixture) has been reported since 1990 (Iwata et al. 1993) and no measurement of endosulfan ever.

We investigated concentrations of POPs in surface seawater along 38°–24° N/28°–67° W in the subtropical North Atlantic with the aim to contribute data to help understanding distributions and long-term and large-scale cycling of these pollutants. The region is a hardly sampled sea region which encompasses a supposedly very clean region, i.e. the Sargasso Sea and the southern arm of the cycle of currents dominating the North Atlantic Gyre (consisting of Gulf Stream, North Atlantic, Canary, North Equatorial and Antilles currents; Harvey and Steinhauer 1974).

## Methodology

### Sampling

**Fig. 1:**
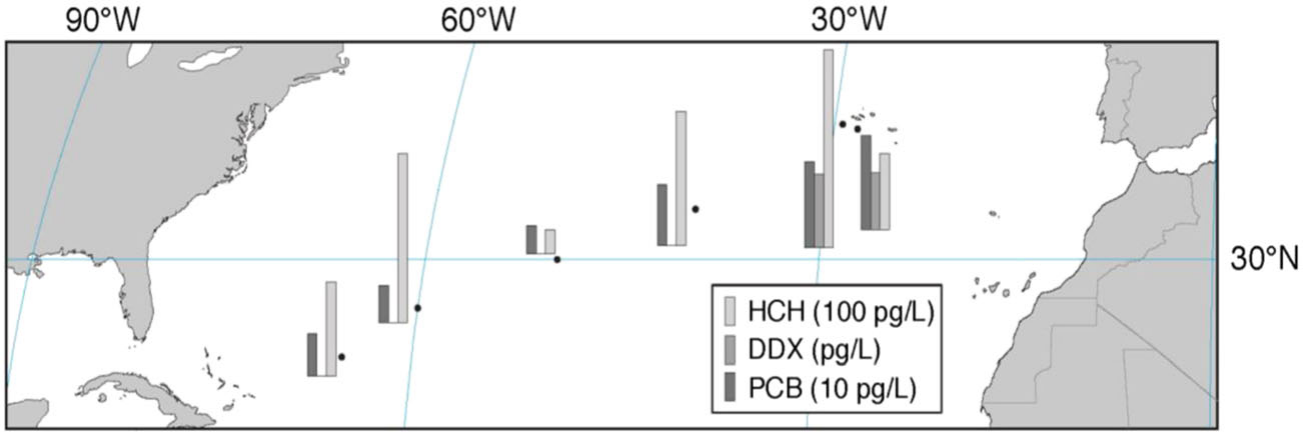
Sampling locations and concentrations of selected pollutants in surface seawater of the North Atlantic

Eight seawater samples were collected, four from the Azores region and four along the transect between Azores and Jamaica (Fig. 1; Table 1; SM, Table S1). Samples were taken only while the ship moved ahead at speeds ≥5 knots.

**Table 1:** Dissolved (including colloidal) phase concentrations in surface seawater of (a.) OCPs and (b.) PCBs (pg L^−1^), sampling location

	Azores region		East-west (40°–67° W) transect			
	37.9° N/28.1° W	38.1° N/29.7° W	33.0° N/40.0° W	30.0° N/50.0° W	27.0° N/60.0° W	24.0° N/66.6° W
a.						
PeCB	3.83	4.96	2.21	3.34	3.56	2.85
HCB	2.27	2.06	3.33	4.93	6.10	4.91
α-HCH	19.1	56.7	38.2	8.31	53.2	27.1
β-HCH	9.49	13.1	8.03	5.23	8.25	6.10
γ-HCH	152	555	444	76.9	557	309
δ-HCH	<0.70	2.19	2.38	<1.30	2.36	1.73
Σ_4_HCH	180	627	492	90.4	621	344
*o,p′*-DDE	<0.49	<0.48	<0.90	<0.91	<0.89	<0.91
*p,p′*-DDE	<2.37	<2.34	<4.39	<4.42	<4.34	<4.42
*o,p′*-DDD	<0.34	<0.34	<0.64	<0.64	<0.63	<0.64
*p,p′*-DDD	<0.27	<0.27	<0.50	<0.51	<0.50	<0.51
*o,p′*-DDT	0.91	0.63	<0.79	<0.79	<0.78	<0.79
*p,p′*-DDT	1.19	1.12	<0.70	<0.70	<0.69	<0.70
Σ_6_DDX	2.10	1.75	<LOQ^a^	<LOQ^a^	<LOQ^a^	<LOQ^a^
Heptachlor	<6.11	<6.03	<11.3	<11.4	<11.2	<11.4
Aldrin	<12.2	<12.0	<22.5	<22.6	<22.2	<22.6
Isodrin	<14.6	<14.4	<27.0	<27.2	<26.7	<27.2
α-Chlordane	4.0	3.9	<5.8	<5.8	<5.8	<5.8
γ-Chlordane	<3.0	<3.0	11.1	<5.8	<5.8	<5.8
α-Endosulfan	8.8	4.5	<5.8	<5.8	<5.8	<5.8
Mirex	<2.74	<2.71	<5.07	<5.10	<5.01	<5.10
b.						
PCB28	4.88	4.53	3.64	2.68	3.86	3.40
PCB52	5.76	4.06	3.06	2.58	2.72	2.71
PCB101	4.91	3.07	4.21	2.40	2.98	3.00
PCB118	1.51	1.21	1.70	0.72	0.78	0.98
PCB153	4.14	4.00	4.62	1.17	2.00	2.66
PCB138	3.70	3.47	4.27	1.30	1.53	2.28
PCB180	<2.31	1.44	0.60	<4.29	<4.22	0.26
Σ_7_PCB	24.9	21.8	22.1	10.8	13.9	15.2

^a^Denoting the sum of the LOQs of the group of individual substances listed

Samples were from unfiltered and untreated seawater that was continuously being pumped throughout the whole cruise from an inlet orifice at the bow of the ship in about 5 m below water surface and discharged at the rear of the ship. The pipes, sieve, inlet valve and other parts’ materials in touch with the seawater were made from ethylene-propylene-diene-monomer (EPDM) or perfluoroalkoxy polymers. Sampling was started after the laboratory tap was open running for at least half an hour. The travel time of the water from inlet through pump to laboratory was 5 min. From the tap, the water was filled directly into a 20 L uncoloured glass jar, which was subsequently sealed with a silicon rubber stopper lined with aluminium foil. The sample was then transferred immediately into a cooling room kept at 10 °C. For protection from light, the glass jars were lined with cardboard to and placed in wooden cages. After the cruise, the samples were transported in an uncooled container carrier to the home laboratory (ca. 20 days).

The samples were filtered (glass fibre filters, previously heated to 450 °C) and confirmed as ‘blue ocean’ samples by dissolved organic carbon (DOC) analysis (0.8–1.5 mg L^−1^) prior to processing (see SM, S1.2 for details). Then, samples were partly pooled (four from the Azores region were pooled into two, leading to in total of six samples; see SM, Table S1), then spiked with an internal standard (50 μL of a solution of PCB congeners not occurring in the environment, i.e. PCB30, PCB185), then extracted in an XAD-2 column (100 mL min^−1^) and eluted with DCM (previously dried over Na_2_SO_4_, 300 mL per sample), then dried over Na_2_SO_4_, evaporated to dryness, and finally dissolved in i-octane (1 mL).

### Chemical analysis

Samples were analysed using a GC-MS/MS Agilent 7890 coupled to Agilent 7000B fitted with a SGE HT-8 column (60 m × 0.25 mm × 0.25 μm; SGE, Victoria, Australia) for seven indicator PCBs (congeners 28, 52, 101, 118, 138, 153, 180), penta- and hexachlorobenzene (PeCB, HCB), α-, β-, γ- and δ-HCH, *o,p′*- and *p,p′*-DDE, -DDD and -DDT (so-called DDX compounds). For the analysis of heptachlor, *trans*- and *cis*-heptachlorepoxide, aldrin, dieldrin, isodrin, chlordecone, methoxychlor and mirex, an Agilent 6890N GC coupled to Waters Micromass Quattro Micro GC was used. The MS was operated in EI+ mode; multiple reaction monitoring (MRM) was selected for analytes quantification (SM, Table S3). EI+ was selected for better stability and linearity of responses. The GC was fitted with an Rxi-5Sil MS column (60 m × 0.25 mm × 0.25 μm; Restek, USA). Injection was splitless 3 μL at 250 °C. He was used as carrier gas at constant flow of 1.5 mL min^−1^, the GC temperature programme was as follows: 80 °C (1 min), 20 °C min^−1^ to 200 °C, 1.5 °C min^−1^ to 260 °C and 50 °C min^−1^ to 310 °C (5 min). PCB121 was used as injection standard for chlorinated substances. The temperature programme was 80 °C (1 min hold), 40 °C min^−1^ to 200 °C and 5 °C min^−1^ to 305 °C. The injection volume was 3 μL in splitless mode at 280 °C, with He used as a carrier gas at constant flow of 1.5 mL min^−1^.

Endrin, α- and γ-chlordane, α- and β-endosulfan and endosulfan sulphate could be analysed more sensitively with a method using a GC-QExactive-Orbitrap-MS operating in full scan from 67 to 1000 mZ. Separation of analytes was performed by Trace 1300 GC using a 30 m TraceGOLD™ column (0.25 mm diameter, 0.25 μm film thickness; ThermoFisher Scientific), operating at an initial temperature of 80 °C and holding for 1 min before increasing to 310 °C at 7 °C per min and holding for 26 min. The injection volume was 3 μL in splitless mode at 220 °C, with He as a carrier gas, 1 mL min^−1^. Fragmentation was by negative chemical ionization (NCI) using methane as the reagent gas at 1.25 mL min^−1^ and resolution set at 60,000 for mZ = 200.

Recovery of native analytes varied 88–103 % for PCBs, 75–98 % for organochlorine pesticides (OCPs). The results were blank (subtracted the mean of two blank values), but not recovery corrected.

Two blank samples were created by filling 1 L of ultrapure (deionized) water into same bottles. Blank samples were shipped and handled in the same way as samples. The instrument limit of quantification (ILOQ) was 0.005–0.021 ng, corresponding to 0.16–1.2 pg L^−1^ for PCB, and 0.008–0.022 ng, corresponding to 0.25–1.3 pg L^−1^ for most OCPs (SM, Table S2). However, ILOQ was 0.09–0.56 ng, corresponding to 2.7–32 pg L^−1^ for heptachlor, *cis*-heptachlorepoxide, aldrin, isodrin, α- and γ*-*chlordane, α-endosulfan and mirex. ILOQs >0.9 ng L^−1^, corresponding to ≈18 pg L^−1^ in the largest sample, applied for *cis*- and *trans*-heptachlorepoxide, dieldrin, endrin, endrin aldehyde and ketone, oxychlordane, β-endosulfan, endosulfan sulphate, chlordecone and methoxychlor (Table S2). As such, levels are beyond expected values for the open ocean (e.g. Iwata et al. 1993; Cai et al. 2010; Kunugi et al. 2010; Lohmann et al. 2012); these analytes are not reported here any further. LOQ was defined as the maximum of the field blank values.

## Results and discussion

### Levels and longitudinal and temporal trends

The determined concentrations (Table 1) refer to the dissolved phase (including colloidal), while the mass fraction associated with suspended and settling particulate matter (> 0.45 μm of size) is excluded (by filtration, above). This particulate fraction is expected to be low in surface waters of the studied sea region during winter: <2 % of HCH and low chlorinated PCBs, <4 % of DDT are predicted to be associated, while 5–13 % is expected for the most lipophilic among the targeted substances, PCB180 (log K_ow_ = 6.9; multicompartment chemistry-transport model with embedded biogeochemical model; Lammel and Stemmler 2012).

#### HCB and PeCB

HCB is a particularly persistent and homogeneously distributed pollutant, at least in the atmospheric environment. However, the spatial variability of HCB in this dataset, characterized by the relative standard deviation σ/μ = 0.41 (0.40 by average for all pollutants), is not less than that of other pollutants (Table 1). The HCB concentrations, 2.1–6.1 pg L^−1^, are within the range of previous findings (Table 2), while PeCB (2.2–5.0 pg L^−1^) had hardly been reported before from Atlantic waters.

**Table 2:** Overview of selected contaminants’ surface seawater concentrations observed in the central (Azores region), and central and western subtropical North Atlantic Ocean (pg L^−1^) and comparison with literature values from regions of (a) the north (N) and (b) the south (S) and Equatorial Atlantic Ocean

Site, year	Σ_7_PCB^a^	HCB	ΣHCH^b^	ΣDDX^c^	Reference
a.					
N Atlc 1989–1990	21–29		80–170	0.7–0.9	Iwata et al. (1993)
N N Atlc 1993	0.3–11.4				Schulz-Bull et al. (1998)
E N Atlc 1999–2000			33–179		Lakaschus et al. (2002)
NE N Atlc 62.5° N 2004		2.5	3.9		Lohmann et al. (2009)
E N Atlc 2005	53 ± 55				Gioia et al. (2008)
E N Atlc 2008			1.8–30		Xie et al. (2011)
W N Atlc 2009	1.2–3.0	0.15–3.0	17–57		Lohmann et al. (2012)
C N Atlc (Azores region) 2015	22–25	2.1–2.3	171–612	1.7–2.1	This work
C&W subtropical N Atlc 2015	11–22	3.3–6.1	85–611	<1.5	This work
b.					
Site, year	Σ_7_PCB^a^	HCB	ΣHCH^b^	ΣDDX^c^	Reference
E S Atlc 1997–98			5.5-25^d^		Jantunen et al. (2004)
E S Atlc 1999–2000			3–12		Lakaschus et al. (2002)
E S Atlc 2005	16 ± 14				Gioia et al. (2008)
E S Atlc 2008			0.3–5.1		Xie et al. (2011)
E&W equat’l Atlc 2009	0.7–3.4	0.10–1.1			Lohmann et al. (2012)

Concentrations <LOQ or detection limit considered equal to zero. Limited number of individual substances included for the sake of comparability

^a^PCB28, -52, -101, -118, -153, -138, -180

^b^Sum of α- and γ-HCH

^c^Sum of DDT and DDE isomers

^d^α-HCH only

#### HCH

The concentration in seawater is high (Table 1a; Fig. 1). HCH has previously been found in the same, the upper pg L^−1^ range in the North Sea, in the northern North Atlantic (>60° N) and in the Arctic Ocean in the 1990s, but <200 pg L^−1^ further south (Iwata et al. 1993; Lakaschus et al. 2002; Table 2) including in the same sea region, the central North Atlantic (Iwata et al. 1993). In 2009, 17–57 pg L^−1^ were measured 10°–16° further north in the western North Atlantic (40° N/71° W; Lohmann et al. 2012). The spatial variabilities of α- and γ-HCH, characterized by σ/μ = 0.57 and 0.59 respectively, are among the highest in this dataset. In 2015, with α-HCH/γ-HCH = 0.09–0.13 (by average 88 and 8.8 % of HCH respectively) the isomer ratio is found remarkably low and is quite stable at all sites (σ/μ = 0.15). This may point to the earlier reversal of air-sea exchange of α-HCH than γ-HCH, related to the historic usage patterns (Jantunen and Bidleman 1995). Trends in K_aw_ (higher for γ-HCH than for α-HCH; Xiao et al. 2004) as well as a possible, yet unconfirmed conversion of the γ-HCH into α-HCH in seawater (Hühnerfuss et al. 1992) would tend to sustain higher isomeric ratios. This isomeric ratio was found higher 15 and 25 years before, α-HCH/γ-HCH = 0.5 ± 0.2 and ≈6 respectively, in surface seawater of the Atlantic (Lakaschus et al. 2002; Iwata et al. 1993), and was 6 years earlier still very high in the western equatorial Atlantic (α-HCH/γ-HCH ≈ 3 ± 1; Lohmann et al. 2012). The latter can be explained by the historically later shift from technical HCH to lindane in countries of the inner tropics as compared to European and North American countries (Li 1999). The level of HCH and the isomeric ratio γ-HCH/α-HCH are surprisingly high. They cannot directly be compared to other findings, as no such data exist. These results are based on quality controlled data and apparently not subject to sampling or analytical errors. β- and δ-HCH, isomers which partition more to water than the other isomers (K_aw_ 1–2 orders of magnitude below the respective values for α-and γ-HCH), account for 2.1 and 0.6 % of HCH respectively. To our best knowledge, this is the first measurement of δ-HCH in open seawater >LOQ.

#### DDX

The concentration of DDT and its metabolites in seawater was mostly <LOQ, but DDT isomers were 1.7–1.9 pg L^−1^ in the Azores region (Table 1a; Fig. 1), hence, a factor of ≈2 higher than 25 years before (0.7–0.9 pg L^−1^; Iwata et al. 1993) and for the isomer *p,p′*-DDT a factor of ≈5 higher than in the mid-latitudes Gulf stream (≈38° N/70° W; Lohmann et al. 2012). Atmospheric deposition is the only source of DDX compounds in the open ocean, as the metabolites DDE and DDD are formed in soils, but not in seawater. On the timescale of decades, past riverine input may be transported into the central Atlantic Ocean, but deep-water formation and partitioning to sinking particles would have transferred the substance to deeper layers (Stemmler and Lammel 2013). To our best knowledge, eventual historic usage of DDT on the Azores was not reflected in environmental contamination of the region (Roscales et al. 2010). However, the Azores region, unlike most of the east-to-west (40°–67° W) transect is in fact located in the very sea region which had historically received a major DDT load through atmospheric depositions (Stemmler and Lammel 2009, 2013). This is supported by studies of marine biota in the region (Magalhães and de Barros 1987). These DDT depositions originated mostly from emissions in the USA, 30°–50° N, during 1955–1975 (Semeena and Lammel 2003, 2009). While most of the pollutant load has sunk to below the thermocline, the direction of air-sea exchange of *p,p′*-DDT in the region is expected to have been net-volatilizational since the 1970s (Stemmler and Lammel 2009 and 2013). As degradation of DDT isomers in seawater is assumed to be negligible (no experimental data available, though), the high isomeric ratio found in this study, *o,p′*-DDT/*p,p′*-DDT = 0.56–0.76 (Table 1a), distinctly higher than in atmospheric deposition (≈0.2 in total deposition in central Europe; Jakobi et al. 2015, and own unpublished data; MONAIRNET project 2011–2012; but *o,p′*-DDT/*p,p′*-DDT > 1 in China; Yue et al. 2011) or in the technical mixture (0.29; Spencer and Cliath 1972), indicates a lower water solubility (or higher K_aw_) of *p,p′*-DDT as compared to *o,p′*-DDT. This would be consistent with *p,p′*-DDT being the more lipophilic isomer and present, deficient knowledge of water solubility and Henry’s law coefficient (Pontolillo and Eganhouse 2001; Rippen 2008; Shen and Wania 2005). Another possible explanation of the high isomer ratio could be atmospheric deposition of DDT which had been released as an impurity of the pesticide dicofol (*o,p′*-DDT/*p,p′*-DDT > 1; Qiu et al. 2005). Dicofol had been recently applied in European countries of the North Atlantic (12 t in Spain in the year 2000, smaller amounts in Portugal and France; Denier van der Gon et al. 2007). However, in the European Union, DDT impurities in marketed dicofol are limited to <0.1 % (Qiu et al. 2005). Because of the rather high LOQs of the metabolites, DDE and DDD (Table 1a), the degree of metabolisation, (DDE + DDD) / DDX <0.62 and <0.89 in the Azores region, is not conclusive.

#### Endosulfan

The α-isomer was detected in the Azores region (8.8 and 4.5 pg L^−1^), but not along the transect (<5.8 pg L^−1^; Table 1), while the β-isomer and the metabolite endosulfan-sulphate, not determined sensitively enough (LOQs at 31–58 pg L^−1^), could not be detected. Endosulfan pollutant had not been reported from the Atlantic Ocean before. The endosulfan concentration found in this study in the North Atlantic 2015 was clearly below the levels found in shell seas of the North Pacific in 2008 (27–98 pg L^−1^ in the Japanese and Okhotsk Seas; Cai et al. 2010) but close to the range reported from the open northwestern North Pacific Ocean in 2010, i.e. 0.1–5.8 pg L^−1^ (Zhong et al. 2012). Differences between the levels in the Atlantic and Pacific oceans could be explained by atmospheric transport patterns between major application areas and the respective open sea regions. In fact, the sites sampled in our study are most likely not in the receptor area for emissions in the southeast of the USA, particularly Florida (a major emission area until 2013; Potter et al. 2014). Prevailing wind directions in Florida are N-NE in winter and S-SE in summer, putting our sites upwind during part of the year. In contrast, the sampling sites in the Pacific Ocean are located in a region which receives much of the outflow of continental East Asia. Endosulfan had also been reported from Arctic Ocean surface seawater at quite high concentrations (Jantunen and Bidleman 1998; Weber et al. 2010; Cai et al. 2010; Zhong et al. 2012), another sea receiving air from polluted (mid latitudes) regions (e.g. Octaviani et al. 2015).

#### Other pesticides

Other pesticides were found <LOQ except α- and γ-chlordane which were found ≈4 and 11 pg L^−1^ at 2 and 1 site, respectively, while <5.8 pg L^−1^ elsewhere (Table 1). Most of these substances had not been reported from the Atlantic Ocean before. However, α- and γ-chlordane ranged 1.8–4.5 and 1.4–2.5 pg L^−1^ respectively, in the North Atlantic on a more southerly east-to-west transect (Iwata et al. 1993). Dieldrin levels were 2–15 pg L^−1^ and isodrin <LOQ-7 pg L^−1^ in the North Sea in 2009–2010 (Mai et al. 2016), consistent with <14 or <27 pg L^−1^ found here (isodrin). Aldrin, dieldrin and endrin have been reported in the mid to upper pg L^−1^ range in shelf seas of the Pacific Ocean (Cai et al. 2010).

#### PCBs

Concentrations of six congeners exceeded LOQ at all sites, and of one congener, PCB180, at 3 out of 6 sites. The patterns were highly correlated across all sites (significant on the *P* > 0.05 level; *t* test), least related to the site at 40° W, which showed minimal mass fractions of low chlorinated and maximal mass fractions of high chlorinated congeners (*P* > 0.05 in two cases). The PCB pollution indicated is within the range spanned by previous measurements in the Atlantic Ocean (Table 2). In comparison with measurements in 2009 in the same region (Lohmann et al. 2012), PCB seem to have increased by a factor of ≈5 at 27° N and by more than a factor of 10 at 24° N in the western N Atlantic (60°–67° W, longitude mismatch is ≈5°, i.e. more westerly in 2015 at 24° N, and more easterly in 2015 at 27° N). Similarly, HCB appears to have increased by approximately one order of magnitude. The measurements, however, were in different seasons, i.e. summer 2009 and winter 2015, with a difference in sea surface temperatures of 2.5–3.2 K, which, in turn, corresponds to a change in Henry’s law coefficient of 25–28 % for PCBs and HCB (Li et al. 2003; Shen and Wania 2005). This suggests that temperature cannot explain the big differences in seawater concentrations. Also, the geographic locations’ mismatch is unlikely to explain the big discrepancy, as both cruises’ routes were in the area of the same section of the North Atlantic Gyre (i.e. the southern arm of the Antilles current). For PCBs, similarly to DDT, the Azores region, more than the east-to-west (40°–70° W) transect is in fact located in the very region of the Atlantic Ocean which had historically received a major load through atmospheric depositions (Lammel and Stemmler 2012).

Recent input by atmospheric deposition remains as a possible explanation for the observed pattern and trend (comparedwith 2009). This cannot be verified by the aforementioned model simulation, as it was not forced by historic meteorology (re-analyses).

Along the east-to-west transect (30° longitudes, corresponding to ≈2500 km), the seawater temperature increased from 20.7 to 25.5 °C. None of the pollutant concentrations behaved inversely correlated as would be suggested by the air-water phase equilibrium of persistent substances in a well-mixed region. Instead, PCBs and HCHs (but not PeCB and HCB) showed even a pronounced minimum at 50° W. This least polluted sampling site is located close to the centre of the North Atlantic Gyre. PeCB and HCB are even found correlated with temperature along 20° longitudes (for the three samples taken 40°–60° W). Earlier, HCB (0.1–1 pg L^−1^ range) was found inversely correlated with sea surface temperature in the eastern and western equatorial Atlantic, but not in the study region (western North Atlantic, 60°–67° W) in 2009 (Lohmann et al. 2012).

### Comparison with multicompartment chemistry-transport modelling

#### Endosulfan

Global cycling of endosulfan can hardly be constrained by modelling, because of the lack of reliable emission estimates. The endosulfan concentration range found in Atlantic surface seawater, <5.8–8.8 pg L^−1^ for α-endosulfan, provides valuable constrainment for model predictions under various emission scenarios (Table S4a); while an upper emission scenario clearly overestimates, a lower emission scenario slightly overestimates the observations. The difference between the two emission scenarios is twofold: Only the top eight agricultures with their reported endosulfan application rates are considered in the lower emissions scenario, while application in agriculture worldwide assuming high application rates (those recommended in the USA) is included in the upper emission scenario (Table S4 for details). The comparison with observations now indicates application rates worldwide may have been mostly lower than in the USA or even lower than reported by those countries which applied most (including USA). The disagreement between observed and predicted data increases if a debated conversion of the endosulfan isomers, i.e. of the α- into the β-isomer (Weber et al. 2010) is modelled (Table S4). This may indicate that the conversion is not occurring in seawater or the equilibrium between the isomers is favouring α-endosulfan against β-endosulfan less than observed in mesocosm experiments (Walse et al. 2003).

#### PCBs

Global PCB distributions 1950–2100 in response to historic emissions have been modelled (Lammel and Stemmler 2012; Octaviani et al. 2015), simulating one possible realization of present and future climate. The model-predicted surface seawater concentration of PCBs (i.e. congeners 28, 101, 153 and 180) in the Azores region and along the transect for 2015 is 6–145 and 0.5–30 pg L^−1^ respectively (Table S4b), hence, overestimates the observations. The agreement is good (within a factor of ≈3; see Table S4b) at the western end of the transect: Unlike the observations, which show a minimum at ≈50 W (Table 1b), in the model PCB, concentrations decrease continuously from east to west by a factor of ≈7. This overestimation in the eastern part of the study area (30°–50° W) could indicate too high model emissions in Europe, which is dominating the input to this sea region (trade wind zone), possibly in combination with underestimated degradation or sinking rates in seawater. Sinking can be ruled out, as it is about oligotrophic seas (low suspended and sinking particle concentration) without significant deep-water formation. The emission estimate used in this model simulation was the maximum estimate of Breivik et al. (2007) for the congeners modelled 40–100 % higher than their minimum estimate. This uncertainty cannot explain more than a minor contribution to the discrepancy in surface seawater concentrations. Due to lack of experimental data, degradation rates input to the model were merely coarse estimates (Wania and Daly 2002).

## Conclusions

The current reported measurements in the subtropical North Atlantic are from sea regions never sampled before for POPs, except at 60°–70° W (in 2009; Lohmann et al. 2012). While the surface seawater concentration levels of PeCB, HCB, PCBs and also DDT are in line with earlier observations in the North Atlantic, HCH is found clearly elevated (Table 2). This observation should be interpreted in the light of future studies covering HCH in surface seawater of the central and eastern subtropical North Atlantic.

East-to-west gradients were obvious. Even within the limited area of the two samples collected in the Azores region (1.6° longitudes, corresponding to ≈150 km), the spatial variability of some pollutants, in particular the HCH isomers (except β-HCH) was very pronounced, as big as along the entire transect. This emphasizes the significance of spatial pollution patterns, driven by historic atmospheric deposition, ocean currents and deep-water formation, rather than mixing and equilibrium for the regional pollutant distribution in surface waters. Discrepancies of the findings for PCB with model predictions point to severe knowledge gaps regarding PCB emissions and degradation in seawater, both currently based on uncertain estimates.

More monitoring of pesticides covering more substances (including so-called emerging pollutants) and using more sensitive methods (for lower LOQ) are needed to understand distributions and cycling of pollutants and assess the success of international chemical legislation (Stockholm Convention on POPs), in particular regarding recent amendments (endosulfan). Apart from geographic gradients, also vertical distributions should be addressed to better account for the 3D distribution of pollutants in the global ocean.

The comparison of the seawater concentrations with model results of long-term and large-scale cycling suggests that estimated degradation rates of PCBs in seawater may be overestimated and that a debated conversion of the endosulfan isomers, favouring the α-isomer (Weber et al. 2010), is not affecting the isomer ratio that much than mesocosm experiments suggest (Walse et al. 2003). More laboratory and close-to-laboratory studies into environmental chemicals’ fate are needed which better mimic the marine environment (salinity, microbial conditions, etc.). Furthermore, the comparison with model data suggests that application of endosulfan in global agriculture was less intensive than reported in the USA, historically one of the top users of this pesticide, and certainly among North Atlantic coastal states. Only with better emission and fate data, the modelling of globally distributed PCBs and endosulfan can be advanced and environmental exposure mapped and quantified.

## Electronic supplementary material

ESM 1 (PDF 215 kb)
