# Supplementary material for: Organochlorine pesticides and polychlorinated biphenyls along an east-to-west gradient in subtropical North Atlantic surface water
Source: Environ Sci Pollut Res Int. 2016 Aug 18;24(12):11045–52. doi: 10.1007/s11356-016-7429-z (PMC5393290; doi:10.1007/s11356-016-7429-z)
Supplement: Supplementary file 1 — (PDF 215 kb) [file 11356_2016_7429_MOESM1_ESM.pdf]

Supplementary material

Organochlorine pesticides and polychlorinated biphenyls along an east-to-west gradient in subtropical North Atlantic surface waters

Gerhard Lammel[1,2*], Alejandro Spitzy[3], Ondřej Audy[2], Sabine Beckmann[3], Garry P. Codling[2], Lisett Kretzschmann[3], Petr Kukučka[2], Irene Stemmler[4]

[1] Max Planck Institute for Chemistry, Multiphase Chemistry Dept., Mainz, Germany, *g.lammel@mpic.de

[2] Masaryk University, Research Centre for Toxic Compounds in the Environment, Brno, Czech Republic

[3] University of Hamburg, Centre for Earth System Research and Sustainability, Institute for Geology, Hamburg, Germany

[4] Max Planck Institute for Meteorology, Hamburg, Germany

S1 Methodology

S1.1 Sampling

Table S1. Sampling parameters during cruise M113, sections I-II, of RV Meteor

Cruise #<br>Sea region	M113-I<br>Azores region		M113-II<br>East-west (40-67°W) transect			
Sample #	I4 + I5 pooled	I6 + I7 pooled	II1	II2	II3	II4
Latitude / longitude	37.9°N/ 28.1°W	38.1°N/ 29.7°W	33.0°N/ 40.0°W	30.0°N/ 50.0°W	27.0°N/ 60.0°W	24.0°N/ 66.6°W
Date<br>Time (UTC)	7 Jan 15<br>19:00-23:00 h	16 Jan 15<br>16:00-18:30 h	28 Jan 15<br>07:41 h	31 Jan 15<br>19:48 h	3 Feb 15<br>22:30 h	6 Feb 15<br>08:19 h
Volume (L)	32.0	32.4	17.3	17.2	17.5	17.2
Salinity (PSU)	36.18	36.15	36.69	37.00	36.63	36.54
Wind (Bf)	4	5	5	5	3	4
T_{air} (°C)	16.5-16.6	15.8-16.3	19.9	20.1	23.9	24.2
T_{water} (°C)	16.7-17.1	16.8-17.7	20.7	22.8	24.8	25.5

S1.2 Organic trace analytical quality assurance parameters

The instrument limit of quantification (ILOQ; Table S2) was based on 3 times the instrument limit of detection, which in turn is based on 3 times the chromatogramme baseline noise level.

Table S2: Instrument limits of quantification (LOQ) and blank values, given as masses and concentrations, the latter for the biggest (32 L) sample

Analyte	iLOQ mass (ng)	iLOQ concentration (pg L^{-1})	Mean blank (ng)
PCB 28	0.00507	0.275	0.019
PCB 52	0.01047	0.908	0.024
PCB 101	0.01949	0.158	0.056
PCB 118	0.01243	0.327	0.037
PCB 153	0.0137	0.609	0.122
PCB 138	0.02092	0.388	0.094
PCB 180	0.01144	0.428	0.071
Pentachlorobenzene	0.00847	0.654	0.000
Hexachlorobenzene	0.01216	0.358	0.019
α-HCH	0.01404	0.265	0.035
β-HCH	0.01669	0.380	0.000
γ-HCH	0.01485	0.439	0.540
δ-HCH	0.02229	0.522	0.000
o,p'-DDE	0.01559	0.464	0.000
p,p'-DDE	0.00805	0.697	0.038
o,p'-DDD	0.01099	0.487	0.000
p,p'-DDD	0.00872	0.252	0.000

(continued)

Analyte	iLOQ mass (ng)	iLOQ concentration (pg L^{-1})	Mean blank (ng)
o,p'-DDT	0.01363	.343	0.000
p,p'-DDT	0.01203	.272	0.000
Heptachlor	0.195	6.11	0.000
trans-Heptachlorepoxide	3.194	99.8	0.000
cis-Heptachlorepoxide	0.630	19.7	0.000
Aldrin	0.389	12.2	0.000
Dieldrin	0.912	28.5	0.000
Endrin	1.00	30.9	0.000
Endrin aldehyde	1.984	62.0	0.000
Endrin ketone	1.026	32.1	0.000
Isodrin	0.467	14.6	0.000
Oxychlordane	1.615	50.5	0.000
γ-Chlordane	0.10	3.10	0.000
α-Chlordane	0.10	3.10	0.000
α-Endosulfan	0.10	3.1	0.000
β-Endosulfan	1.00	30.9	0.000
Endosulfan sulfate	1.00	30.9	0.000
Chlordecone	20.79	650	0.000
Methoxychlor	1.290	40.3	0.000
Mirex	0.088	2.74	0.000

Table S3: Ions monitored for the MS analysis of target substances on (a) GC-MS/MS Agilent 7890 coupled to Agilent 7000B, (b) Agilent 6890N GC coupled to Waters Micromass Quattro Micro GC (EI+ mode) and (c) GC-QExactive-Orbitrap-MS (CI- mode)

a.

Analyte	Quantification transition	Collision energy (eV)	Verification transition	Collision energy (eV)	t_R (min)
PeCB	252>215	22	250>215	22	11.10
α-HCH	219>183	6	181>145	13	15.73
HCB	285.8>213.8	39	283.8>248.9	18	16.28
γ-HCH	219>183	6	181>145	13	18.58
β-HCH	219>183	6	181>145	13	18.87
δ-HCH	219>183	6	181>145	13	21.95
ε-HCH	219>183	6	181>145	13	22.79
PCB28	258>186	30	256>186	30	23.02
PCB52	291.9>219.9	33	289.9>219.9	33	25.78
o,p‘-DDE	318>248	24	246>176	33	32.09
PCB101	327.9>255.9	33	325.9>255.9	33	32.71
p,p‘-DDE	318>248	24	246>176	33	34.27
o,p‘-DDD	237>165	22	235>165	22	35.10
o,p‘-DDT	237>165	22	235>165	22	36.70
PCB118	327.9>255.9	33	325.9>255.9	33	36.98
p,p‘-DDD	237>165	22	235>165	22	37.49
PCB153	361.8>289.9	33	359.8>289.9	33	37.78
p,p‘-DDT	237>165	22	235>165	22	39.01
PCB138	361.8>289.9	33	359.8>289.9	33	39.22
PCB180	395.8>325.9	33	393.8>323.9	33	42.37

b.

Analyte	Quantification transition	Collision energy (eV)	Verification transition	Collision energy (eV)	t_R (min)
Heptachlor	272 > 237	32	272 > 141	12	16.81
trans-Heptachlorepoxide	353 > 282	12	353 > 253	16	20.82
cis-Heptachlorepoxide	353 > 253	16	353 > 282	12	21.06
Aldrin	263 > 193	24	263 > 228	18	18.66
Dieldrin	263 > 193	22	277 > 207	20	25.15
Isodrin	193 > 157	18	263 > 193	24	20.32
Oxychlordane	387 > 263	14	387 > 287	16	20.81
Methoxychlor	227 > 141	30	227 > 169	24	36.28
Mirex	272 > 237	12	274 > 239	12	40.76

c.

Analyte	Quantification Ion	Verification Ions	t_R (min)
Endrin	269.9414	271.9384, 273.9356	23.48
γ-Chlordane	299.8656	301.8627, 297.8685	21.83
α-Chlordane	265.9045	241.9043, 267.9019	22.13
α-Endosulfan	265.9045	263.9075, 267.9018	22.20
β-Endosulfan	241.9042	403.8169, 239.9067	23.68
Endosulfan sulfate	383.8360	96.9592, 385.8331	24.78

S1.3 Dissolved organic carbon (DOC) analysis

DOC concentrations of all water samples were measured using a high temperature catalytic oxidation analyser (Shimadzu TOC-V) with a Pt catalyst at 680°C. All generated aqueous samples for DOC analysis were acidified to pH ≈ 2 with 85% H_3PO_4 and purged for 5 min to remove inorganic carbon prior to analysis. Synthetic air was used as a carrier gas in the TOC analyser. Standards (potassium biphthalate) were analysed immediately prior to and after analysis of 10 samples and were prepared with ultrapure water from a Microlab-Genpure system (TKA, Germany). The detection limit was found at 0.02 mg L^{-1}. All samples were analysed in triplicate. Precision, in terms of the relative standard deviation, was better than 2%.

S2 Discussion - Comparison with model predictions of surface seawater concentrations

Table S4: Dissolved phase concentrations in surface seawater of (a.) endosulfan and (b.) PCBs (pg L^{-1}) for January-February 2015, predicted by multicompartment chemistry-transport modelling, global multidecadal simulations starting 1950, ≈3° horizontal resolution (Lammel and Stemmler 2012; Octaviani et al. 2015, and unpublished model data).

a.

	Azores region	East-west (40-67°W) transect
	38°N/28-30°W	24-33°N/ 40-67°W
α-endosulfan low emissions (1)	25-28	16-43
α-endosulfan high emissions (2)	75-85	55-150
α-endosulfan high em. (2) conversion (3)	105-119	126-740

b.

	Azores region	East-west (40-67°W) transect
	38°N/28-30°W	24-33°N/ 40-67°W
PCB28 (4)	5.8-12.5	0.55-4.7
PCB101 (4)	19-35	1.4-13.8
PCB153 (5)	50-145	4-30
PCB180 (4)	21-24	1.5-10.6

(1) based on top 8 usage countries only and reported application rates (application to crops; FAO 2009)

(2) based on application to crops worldwide (FAO 2009) using application rates reported from USA (USDA 2012) for most countries

(3) assumes conversion of β- into α-endosulfan in seawater (Weber et al. 2010; Walse et al. 2003)

(4) PCB153 emissions input; model output scaled for historical emissions of this congener (Breivik et al. 2007)

(5) Maximum emission estimate of Breivik et al., 2007.

References

Breivik K, Sweetman A, Pacyna JM, Jones K.C (2007) Towards a global historical emission inventory for selected PCB congeners – a mass balance approach. 3. An update. Sci Tot Environ 377:296-307

FAO (2009) UN Food and Agricultural Organisation, FAOSTAT (crops), URL: http://faostat.fao.org

Lammel G, Stemmler I (2012) Fractionation and current time trends of PCB congeners: Evolvement of distributions 1950-2010 studied using a global atmosphere-ocean general circulation model. Atmos Chem Phys 12:7199-7213

Octaviani M, Stemmler I, Lammel G, Graf HF (2015) Atmospheric transport of persistent organic pollutants to and from the Arctic under present-day and future climate. Environ Sci Technol 49: 3593-3602

USDA (2012) National Agricultural Statistics Service, US Dept of Agriculture, http://quickstats.nass.usda.gov, URL: http://quickstats.nass.usda.gov/results/D2B6AB43-21CC-3DDD-AAF1-CD8FC1AE73A1

Walse SS, Scott GI, Ferry JL (2003) Stereoselective degradation of aqueous endosulfan in modular estuarine mesocosms: formation of endosulfan γ-hydroxycarboxylate. J Environ Mon 5:373-379

Weber J, Halsall CJ, Muir DCG, Teixeira C, Small J, Solomon K, Hermanson M, Hung H, Bidleman TF (2010) Endosulfan, a global pesticide: A review of its fate in the environment and occurrence in the Arctic. Sci. Total Environ. 408:2966-2984
